# A New Insight into the Comonomer Effect through NMR Analysis in Metallocene Catalysed Propene–*co*–1-Nonene Copolymers

**DOI:** 10.3390/polym11081266

**Published:** 2019-07-31

**Authors:** Qiong Wu, Alberto García-Peñas, Rosa Barranco-García, María Luisa Cerrada, Rosario Benavente, Ernesto Pérez, José Manuel Gómez-Elvira

**Affiliations:** 1 School of Engineering, Hong Kong University of Science and Technology, Clear Water Bay, 999077 Kowloon, Hong Kong; 2 College of Materials Science and Engineering, Shenzhen Key Laboratory of Polymer Science and Technology, Guangdong Research Centre for Interfacial Engineering of Functional Materials, Nanshan District Key Laboratory for Biopolymers and Safety Evaluation, Shenzhen University, Shenzhen 518060, China; 3 Key Laboratory of Optoelectronic Devices and Systems of Ministry of Education and Guangdong Province, College of Optoelectronic Engineering, Shenzhen University, Shenzhen 518060, China; 4 Instituto de Ciencia y Tecnología de Polímeros (ICTP-CSIC), Juan de la Cierva 3, 28006 Madrid, Spain

**Keywords:** polypropene copolymers, comonomer effect, metallocene catalysts, microstructure, NMR analysis

## Abstract

The “comonomer effect” is an intriguing kinetic phenomenon in olefin copolymerization that still remains without a detailed explanation. It typically relates to the rate of enhancement undergone in ethylene and propene catalytic polymerization just by adding small fractions of an alpha-olefin. The difficulty lies in the fact that changes caused by the presence of the comonomer in reaction parameters are so conspicuous that it is really difficult to pin down which of them is the primary cause and which ones are side factors with marginal contribution to the phenomenon. Recent investigations point to the modification of the catalyst active sites as the main driving factor. In this work, the comonomer effect in the metallocene copolymerization of propene and 1-nonene is analysed and correlated to the comonomer role in the termination of the chain-growing process. The associated termination mechanisms involved furnish most of chain-free active sites, in which the selective interaction of the comonomer was proposed to trigger the insertion of monomers. A thorough characterisation of chain-end groups by means of the ^1^H NMR technique allows for detailing of specific transfer processes, ascribed to comonomer insertions, as well as evidencing the influence of the growing chain’s microstructure over the different termination processes available.

## 1. Introduction

Incorporation of small quantities of a comonomer, mostly an α-olefin, into ethylene or propene polymerizations usually enhances the kinetics of the process. This is what is known as the comonomer effect. The phenomenon has been reported in Ziegler-Natta, Phillips, and metallocene catalysts with a large variety of comonomers [1,2,3,4,5,6,7,8,9,10,11,12,13,14,15,16,17,18,19,20,21,22,23,24,25,26,27,28,29,30,31,32,33,34] and great concern exists in explaining its true nature, since it has important consequences from a production point of view [16].

Much effort has been devoted to understanding the reasons for this surprising kinetic change, which seems to depend on every copolymerization parameter. Diverse variables have been claimed as driving ones, however the fact is that, to date, there is no definite explanation. A brief overview of them is presented next.

Many authors have claimed solubility enhancement of copolymer chains and subsequent improvement in mass transfer processes as the key factor in Ziegler-Natta and supported metallocene catalysts [2,7,15,22,24,25,26,31,34,35,36,37,38,39,40,41,42,43], however although an easier diffusion of the monomer through less crystalline phases may contribute to a rate enhancement, no satisfactory correlation has been found with crystallinity degree or copolymer solubility [44].

Most explanations are, however, focussed on the modification of both the quality and concentration of active catalyst sites. In fact, many works agree with this assumption as a crucial piece in the puzzle [5,10,11,12,15,21,23,24,27,29,45,46,47,48,49]. For example, fracturing of inorganic particles and polymer aggregates in heterogeneous and homogeneous catalysis, respectively, has been claimed to exert a primary role [1,2,34,36], however the fact that the comonomer effect has been observed in systems both without particle fragmentation and completely dissolved suggests that this factor is not essential.

Another proposed explanation is related to an increment of active sites by activation of dormant species. This would be possible through displacement of the equilibrium set by inactive catalyst-donor complexes towards active catalyst-α-olefin ones [5,20,50,51,52,53,54].

As far as the active site’s quality is concerned, Sacchi et al. explored the modification of the metallocene shielding by changing solvent polarity and co-catalyst bulkiness [49,55]. They found that if the active site was somehow unshielded, the kinetics of the comonomer insertion sped up and the activity improved.

Another factor influencing the active site accessibility is comonomer coordination itself, which has been suggested to enhance spacing between the metal atom and the counteranion. This was proposed by some authors [10,29,32], who suggested that the polymerization rate would be higher for small comonomers due to their inferior steric hindrance. The role of comonomer as a solvating molecule of the active site has also been highlighted by Kryzhanovskii et al. [19].

More recently, some works on Cr(II) Phillips type catalysts have shown that modification of the active centre through selective interaction with α-olefin does occur, and this must be a determinant for a rate enhancement [4,44]. These results support the “trigger mechanism” [12], which proposes the simultaneous coordination of two monomers at the active site, one of them being selectively occupied by the α-olefin, which would play as the trigger species.

Difficulties encountered in the search of a conclusive explanation arise from the fact that most reaction parameters are somewhat altered and it is not easy at all to determine whether the observed kinetic enhancement is mainly dependent on a single factor or on the cooperative contribution of a number of them. However, a certainty does arise from all these works: namely, it is quite plausible that modifications undergone by the catalyst active centre are mostly involved, with the possible assistance of other factors.

Notwithstanding the above detailed concurrence of factors, there is a common denominator for almost all cases. The molecular weight of the copolymer is lower than the one observed for the homopolymer, since the comonomer seems to influence not only propagation rate, but chain transfer as well [3,7,9,29,56,57]. The immediate consequence is that the steady state concentration of chain-free initiating sites is higher and that might enhance the copolymerization kinetics if it is assumed that the specific interaction of initiating metallocene sites with the comonomer triggers the first insertion [44]. Some previous results correlating catalyst activity and chain-transfer termination mechanisms in the copolymerization of propene with 1-pentene support such a hypothesis [57].

In this work, the metallocene copolymerization of propene with 1-nonene is studied at different temperatures. The correlation between catalyst activity and chain transfer efficiency of inserted comonomers is analysed, as well as termination mechanisms lying behind, in order to check the correlation previously found in poly(propylene–*co*–1-pentene) copolymers.

## 2. Materials and Methods

### 2.1. Synthesis of Copolymers and Homopolymers

The homo and copolymerization reactions were performed under inert conditions after careful purification of the initial reactants. Toluene (Merck) and 1-nonene (C_9_, Acros) were dried by refluxing over metallic sodium and further distillation under nitrogen. Nitrogen (Praxair 3X) and propylene (Praxair 2.5) were purified by flowing through oxygen-trap columns and molecular sieves before using. Ethanol and HCl employed were Aroca, 96%, and VWR, 37%, respectively. The metallocene catalyst *rac*-dimethylsilylbis(1-indenyl) zirconium dichloride (Me_2_Si(C_9_H_6_)_2_ZrCl_2_) was supplied by Strem, and the co-catalyst MMAO was supplied by Aldrich as a solution in toluene (MMAO-12, 7 wt. % in Al).

Polypropene homopolymers and poly(propene–*co*–1-nonene) copolymers were prepared in a Büchi glass ecoclave (500 mL) at polymerization temperatures (*T*_pol_) of −5, 10, 25, 40, and 60 °C in 250 mL toluene and with an initial propene pressure of 1.5 bar. Runs were carried out at 1-nonene feeding molar fractions ranging between 0 and 0.34. The reactions were started by incorporation of the metallocene precursor, which was previously activated with the cocatalyst. The final (Al)/(Zr) molar ratio was 1925. The reaction was finished at a 10% propene consumption by adding 5 mL of ethanol. Subsequently, samples were precipitated with a mixture of ethanol and HCl (30:1). The so-precipitated polymers were stirred overnight, filtered, washed with ethanol, and finally dried under vacuum at room temperature.

Poly-1-nonene homopolymers were also obtained in toluene solution by following an identical procedure and conditions to those used for the copolymerizations. In this case, 0.09 moles of C_9_ were injected into the reactor and a pressure of 1.5 bar of nitrogen was used instead of propene. The samples were isolated by removing the toluene in a rotary evaporator, then dissolving with 1-pentene and extracting residues from MMAO and catalyst with acidified water. Finally, after washing with pure water, the 1-pentene and the unreacted 1-nonene were removed by evaporation under vacuum at 120 °C.

Volumes of C_9_ incorporated into the reactor were directly used to calculate feeding molar fractions without performing any correction due to the temperature. It was checked that the comonomer loss in the solution was negligible in all cases, according to the corresponding vapour pressure at the different tested polymerization temperatures, *T*_pol_ [58], and was calculated from Raoult′s law. As for the propene, the molar fraction in the reaction medium was estimated by means of the relationship given by Ferreira et al. for solutions in toluene [59].

### 2.2. Identification of Samples

Copolymer samples will hereinafter be named as C9PP, followed by the polymerization temperature and the closest integer value for the C_9_ mol % content. For instance, C9PP-5_2 stands for a copolymer obtained at −5 °C, of which 1-nonene content is 2 mol %. In the case of homopolymers, samples were simply identified as PP for polypropylene and as polyC_9_ for poly-1-nonene. Similarly to copolymers, the corresponding polymerization temperature was indicated next. With this, PP40 and polyC_9_40 stand, respectively, for polypropylene and poly-1-nonene samples synthesised at 40 °C.

### 2.3. NMR Analysis

^1^H and ^13^C NMR experiments as well as ^13^C-DEPT, ^1^H-^13^C HSQC, and ^1^H-^1^H TOCSY were carried out at 80 °C with a Bruker Avance III HD-400 spectrometer (Billerica, MA, USA) from solutions of the samples in 1,1,2,2-tetrachloroethane-*d*_2_ (70 mg/0.7 mL). The only exceptions were the PP samples, of which ^1^H and ^13^C NMR spectra were obtained with a Bruker Avance III/500 (125.76 MHz) at 100 °C. ^1^H NMR spectra of the samples were performed by accumulating 64 scans. The proton analysis was always carried out prior to the ^13^C one, so as to avoid the appearance of signals caused by processes associated with long residence times of the ^13^C NMR analysis, like degradation and isomerisation. Chemical shifts of ^1^H signals were referred to that of the 1,1,2,2-tetrachloroethane-*d*_2_ at 6.00 ppm.

2D ^1^H-^1^H TOCSY spectra were obtained by performing 16 scans with a delay time of 2 s, an acquisition time of 0.256 s, a spectral width of 3997 Hz in both dimensions, 1024 data points in F1 and F2, a mixing time of 80 ms, and a 90° pulse width (12 µs).

^13^C NMR spectra were recorded with broad band proton decoupling using a 24,038 Hz spectral window, an acquisition time of 1s, a relaxation delay of 5 s, and a pulse angle of 45° (5 μs). A minimum of 8000 scans were recorded. Chemical shifts for ^13^C signals were referred to those of the 1,1,2,2-tetrachloroethane-*d*_2_ at 74.00 ppm.

A 5 s delay time was found to be adequate to assure complete relaxation of ^13^C spins. It was checked by performing the ^13^C NMR spectrum of the highest comonomer content sample (CP60_12) with a 10 s delay time. Differences observed in microstructure, both in composition and in configuration, were within the experimental error (see Table 1 in Results and Table S1 provided in Supplementary Materials).

^13^C DEPT NMR for one of the poly-1-nonene samples was obtained using the Bruker deptsp135 sequence, pulse widths of 10 and 14 μs for ^1^H and ^13^C, respectively, a spectral window of 16,129 Hz, and accumulating 10,000 scans.

2D ^1^H-^13^C DEPT-HSQC spectra were performed using a 90° pulse (14 and 10 μs for ^1^H and ^13^C, respectively), 6.39 MHz spectral width in the ^1^H dimension and 16.59 MHz in the ^13^C dimension, 0.2 s acquisition time, and 2 s relaxation delay.

### 2.4. Size Exclusion Chromatography (SEC)

The SEC analysis of PP samples was performed in a Waters GPC/V 2000 (Milford, MA, USA) equipped with refractive index and viscometer detectors. A set of three columns of the PL Gel type was used with 1,2,4-trichlorobenzene at 145 °C as the solvent and 1 mL/min flow rate. The injection volume was 200 µL and the equipment was calibrated with narrow molecular mass distribution standards of polystyrene.

### 2.5. Differential Scanning Calorimetry (DSC)

The melting temperatures (*T*_m_) were measured in a TA Q100 Instrument calorimeter (New Castle, DE, USA) connected to a cooling system and calibrated with indium and zinc. The samples were heated from −45 °C to either 160 °C for the copolymers or 180 °C for the PP samples, at a heating rate of 10 °C·min^−1^. They were subsequently cooled down to −45 °C, also at 10 °C·min^−1^, and finally heated up again to 160 or 180 °C. The values of T_m_ were taken from the maximum of the endotherm peak obtained after the second heating scan. The results are shown in Table 1.

## 3. Results

### 3.1. Microstructure and Molecular Weight

**Figure 1:**
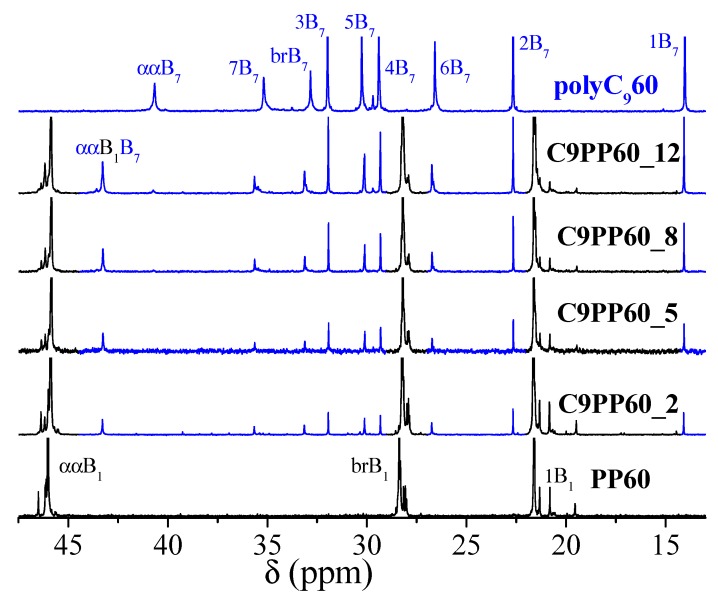
^13^C NMR spectra for the samples obtained at 60 °C.

**Scheme 1:**
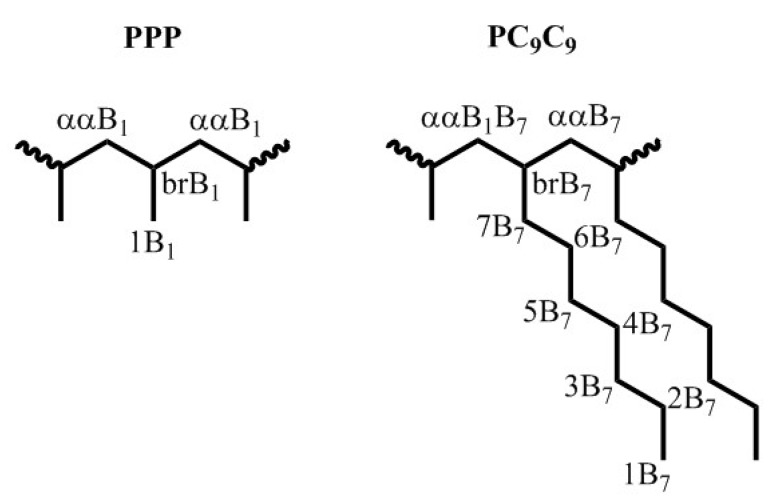
Nomenclature of carbon nuclei in the homo-propene triad (PPP) and in a copolymer triad (PC_9_C_9_).

The microstructure in the composition of copolymers was obtained from the analysis of ^13^C NMR spectra. Assignments were made according to literature [60,61] and they are shown in Figure 1 for the particular case of samples obtained at 60 °C. They were identified in the figure using the established nomenclature for branched polyolefin copolymers [62]. The corresponding identifiers for the different carbon nuclei are shown in Scheme 1.

The relative molar content of 1-nonene (C_9_) of the different copolymers was estimated from the integrals of the methyl signals 1B_7_ and 1B_1_. No significant differences were found between these so-calculated values and those ones obtained from the relationship between methine integrals (signals brB_7_ and brB_1_). The comonomer content of the samples is reported in Table 1.

Additionally, the reactivity ratio product (r_1_.r_2_) was calculated from the comonomer distribution at the diad level using appropriate relationships between ^13^C NMR integrals [6]. The results are also listed in Table 1 and show that r_1_·r_2_ values are not far away from unity. This allows us to confirm that the samples are random copolymers.

Finally, the tacticity of propene sequences was estimated from the methyl region (1B_1_ in Scheme 1: 19.4–22.0 ppm) at the pentad level [60]. The isotactic average length of propene sequences (*n*_1_) was assessed by taking into account any kind of isotactic sequence interruption, either C_9_ insertion or both stereo and regio-misinsertions of propene [63].

Results are summarized in Table S1, provided as supplementary data. This table reveals that propene isotacticity, tracked by means of (mmmm) pentads, decreases as *T*_pol_ rises from –5 to 60 °C.

**Figure 2:**
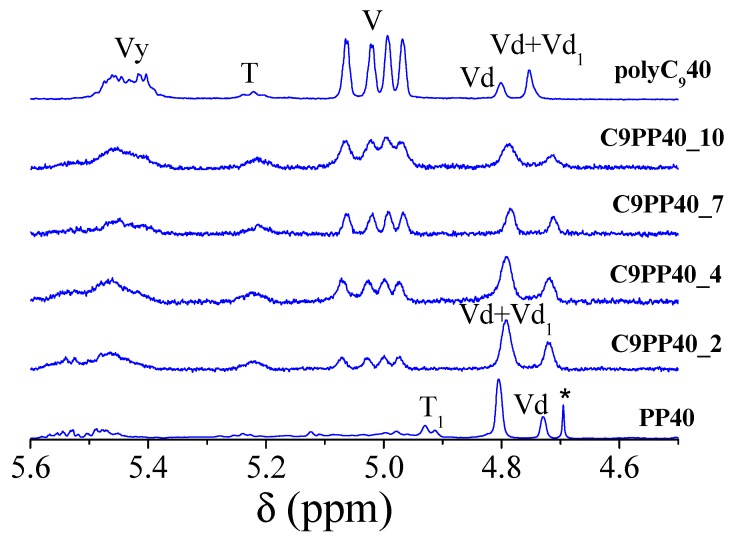
Olefinic window of ^1^H NMR spectra for samples obtained at 40 °C. (* solvent impurity).

**Scheme 2:**
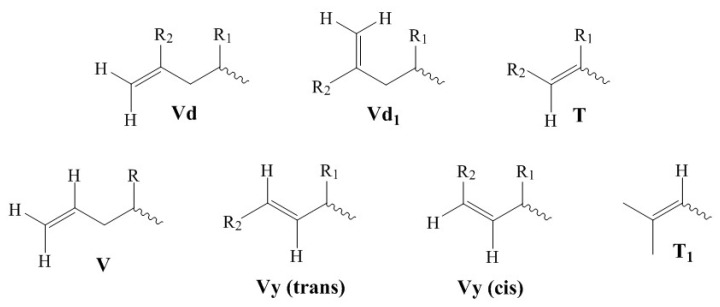
Chain-end groups.

The qualitative and quantitative analysis of chain-end double bonds was performed by ^1^H NMR. It revealed different olefin species, which are shown in Figure 2 for samples obtained at 40 °C as an example. The species displayed are vinylidenes from 4.65 to 4.85 ppm (*Vd* and *Vd*_1_), vinyls from 4.9 to 5.12 ppm, and from 5.78 to 5.95 ppm (*V*), trisubstituted olefins from 5.12 to 5.30 ppm (*T* and *T*_1_) and vinylenes from 5.30 to 5.62 ppm (*V*_y_). The assignment of every signal is indicated in the picture by the corresponding acronym (see Scheme 2) and it can be seen that isobutenyl groups (*T*_1_) are only found in the PP homopolymer.

**Figure 3:**
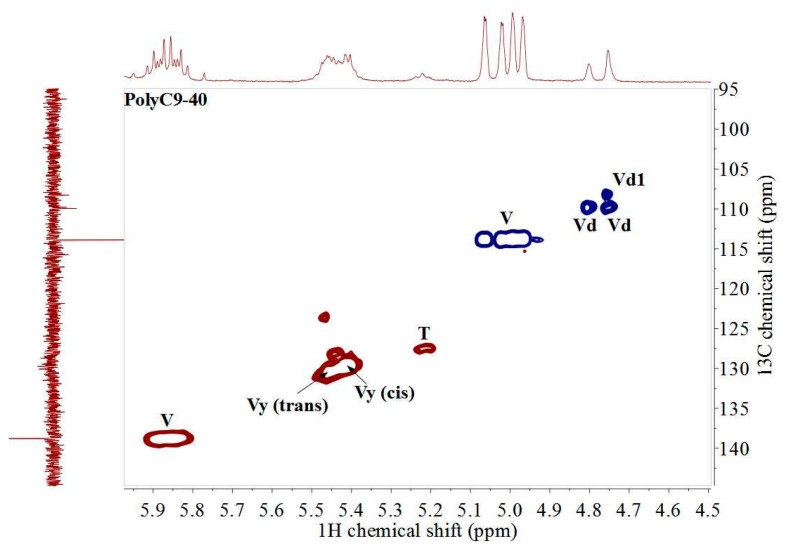
^1^H-^13^C DEPT-HSQC spectrum of the sample polyC_9_40.

As far as *Vd*_1_ olefins are concerned, their protons overlap with the low-field signal of the *Vd* doublet [64,65,66] in the case of PP and C_9_ copolymers (see the higher intensity of the *Vd* + *Vd*_1_ signal in copolymer samples in Figure 2). Consequently, it is the high-field signal of *Vd* groups that yields the content of these species, and it has been subtracted from the *Vd* + *Vd*_1_ integral to estimate the population of *Vd*_1_ olefins. The situation seems to be different in the poly-1-nonene (top ^1^H NMR spectrum in Figure 2) because the asymmetry shown by both *Vd* signals is just the opposite. The ^1^H-^13^C DEPT-HSQC spectrum of poly-1-nonene obtained at 40 °C (represented in Figure 3) confirms the overlapping of two different olefinic protons at 4.75 ppm, which are ascribed to terminal and internal vinylidenes species.

Molecular weights were calculated from the relationship between ^1^H NMR integrals corresponding to protons of main-chain methines and olefin groups, according to the next expression:

$$Mn=\frac{\int_{1.42}^{1.75}\left(CH\right)d\delta \;}{1/2\left[\int_{4.94}^{5.11}\left(V\right)d\delta \;+\int_{5.33}^{5.60}\left(Vy\right)d\delta \;\right]+\int_{4.67}^{4.76}\left(Vd\right)d\delta \;+\int_{5.15}^{5.27}\left(T\right)d\delta \;+\int_{4.87}^{4.94}\left(T_{1}\right)d\delta \;}\times \left[126\cdot f\left(C_{3}\right)+42\cdot f\left(C_{9}\right)\right]$$

where *Vd*, *V*, *V*_y_, *T*, and *T*_1_ correspond to the intensity of the NMR signal for the different chain-end double bonds, according to acronyms given in Scheme 2, and *f*(C_3_) and *f*(C_9_) correspond to molar fractions of propene and 1-nonene in the copolymer, respectively. Integrals were performed on the indicated chemical shift ranges (δ), given in ppm.

It was assumed that metallocene polymerization yields only one terminal double bond every chain [64]. Therefore, all olefin species were taken for such calculus, except for in-chain vinylidenes (see *Vd*_1_ groups in Scheme 2). The values are collected in Table 1.

### 3.2. Evolutions of C_9_ Insertion, Catalyst Activity, and Molecular Weight with the C_9_ Feeding Fraction

**Figure 4:**
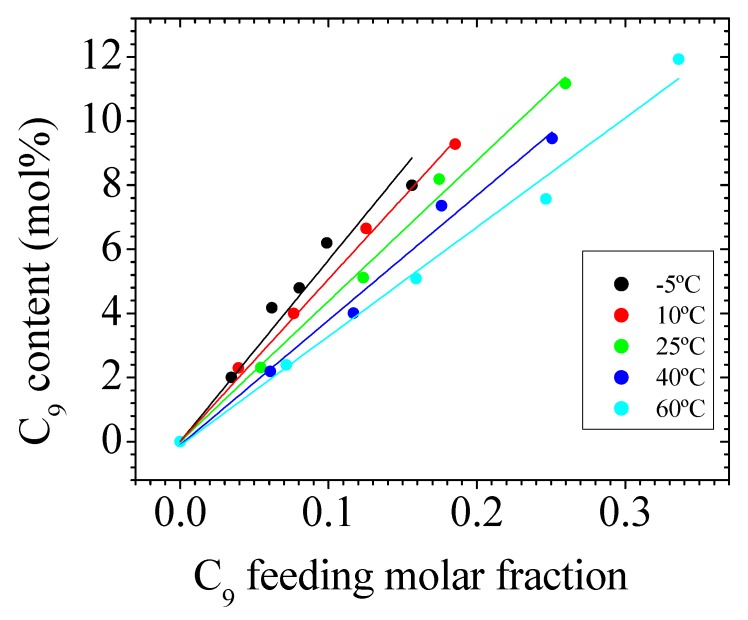
Variation of the C_9_ content in the copolymers with the C_9_ feeding molar fraction at the different *T*_pol_. A colour code is inserted into the figure.

The insertion of C_9_ into PP chains seems to depend on the polymerization temperature, as can be deduced from Figure 4. This graph shows how the copolymer is enriched in C_9_ as long as *T*_pol_ decreases. For instance, at a C_9_ feeding fraction of 0.15, the difference in C_9_ content already fetches about 4 mol % between copolymerizations performed at 60 and –5 °C.

**Figure 5:**
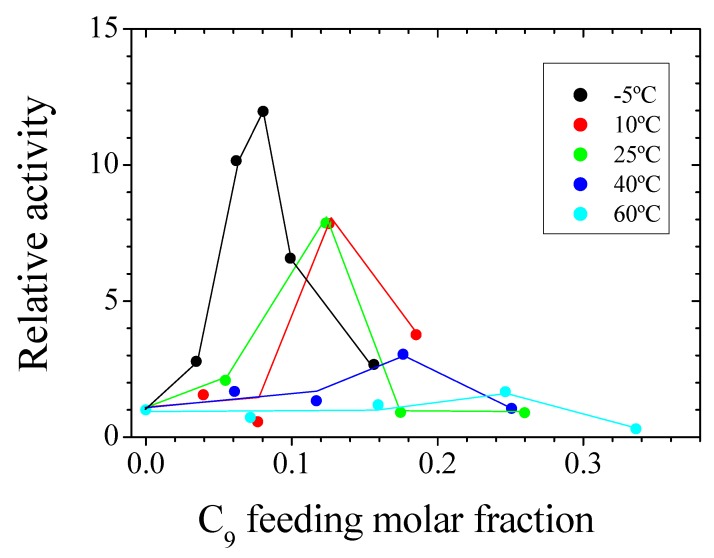
Evolution of relative activities with the C_9_ feeding molar fraction. Polymerization runs are distinguished by the colour code inserted.

The activities for the different copolymerization runs are collected in Table 1, where it can be observed that values increase with *T*_pol_ and go through a maximum in every series. Even though this rise with temperature is expected, the detailed analysis in Figure 5 unravels an aspect that aids in understanding the comonomer effect. As a matter of fact, the relative activities, calculated by taking the corresponding propene homopolymerization′s activity as a reference, make evident that copolymerization kinetics depend on *T*_pol_. Furthermore, the figure first shows that the maximum appears at lower C_9_ feeding ratios as *T*_pol_ decreases. Secondly, the phenomenon is relatively much more conspicuous as the temperature diminishes.

**Figure 6:**
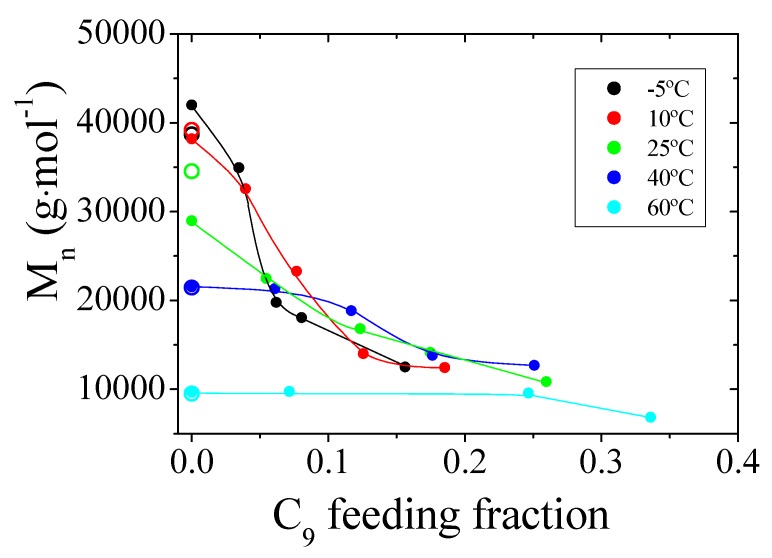
Variation of the number average molecular weight (*M*_n_) with the C_9_ feeding molar fraction. Open symbols: *M*_n_ from SEC; solid symbols: *M*_n_ from ^1^H NMR. A colour code is inserted into the figure.

The kinetic enhancement undergone by the copolymerization, due to the presence of C_9_ in the medium, is also associated with diminution of molecular weights, as is shown in Figure 6. It is evident from this graph that chain size decreases with C_9_ insertion and that the reduction is more effective at lower *T*_pol_. In other words, C_9_ must be involved in a chain shortening process, which seems to be promoted when *T*_pol_ decreases.

### 3.3. Evolution of Olefin Species with the C_9_ Feeding Fraction

**Figure 7:**
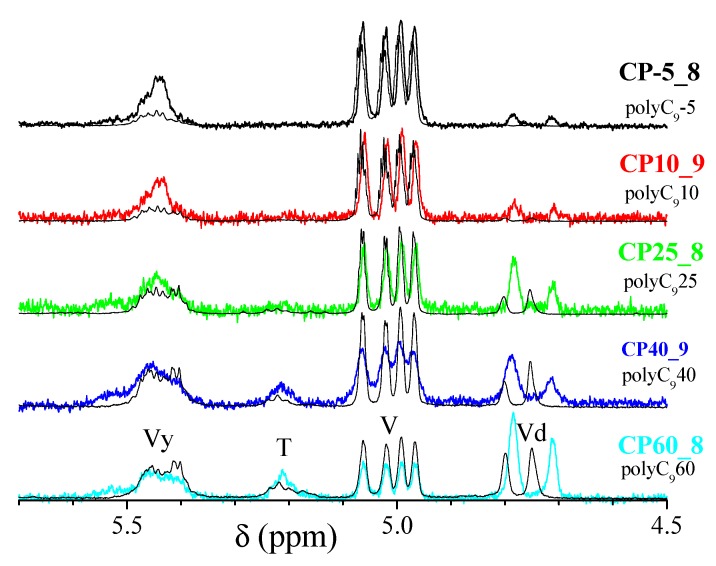
Olefinic window of ^1^H NMR spectra for samples with comparable C_9_ contents, obtained at different *T*_pol_. Spectra of corresponding polyC_9_ samples are shown for comparison.

The relative contribution of every terminal olefin was found to depend on *T*_pol_ rather than on copolymer composition. This fact is illustrated in Figure 7, which shows the ^1^H NMR window under study for copolymers of comparable compositions obtained at different *T*_pol_. It is quite evident that, firstly, important differences arise not only from the relative intensities of signals, but also from the comparison of the *V*_y_ contours; and, secondly, that *V*_y_ profiles match analogous signals in their corresponding polyC_9_ samples. The evolution of all signals will be comparatively tracked next in order to assess the relative importance of the different termination pathways involved.

**Figure 8:**
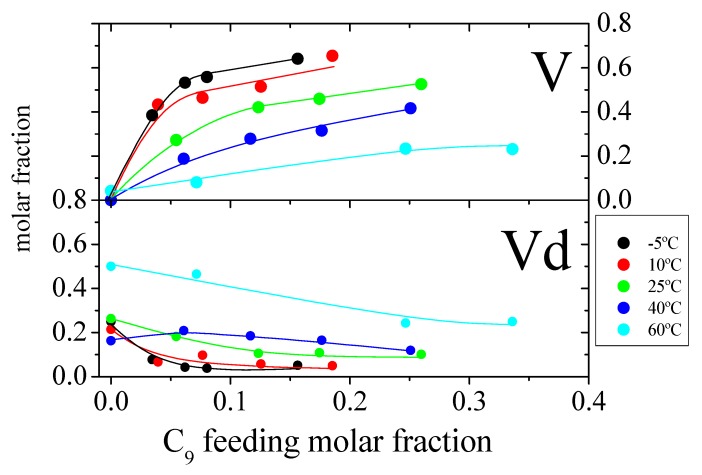
Evolution of *Vd* and V contents with the C_9_ feeding molar fraction for the different series.

As far as vinylidenes are concerned, the evolution with C_9_ feeding fraction depends on *T*_pol_ according to trends presented in the bottom plot of Figure 8. The relative magnitude of the reduction goes along with the selectivity found for the interaction of the catalyst towards C_9_ comonomer, and it is increasingly pronounced as *T*_pol_ decreases. The most important result is that these species come exclusively from propene units. It can be remarked, indeed, in Figure 7 that characteristic *Vd* signals of poly-1-nonene samples are completely absent in any copolymer. This specificity of the copolymer chain-growing termination has interesting mechanistic implications, which will be discussed later.

**Figure 9:**
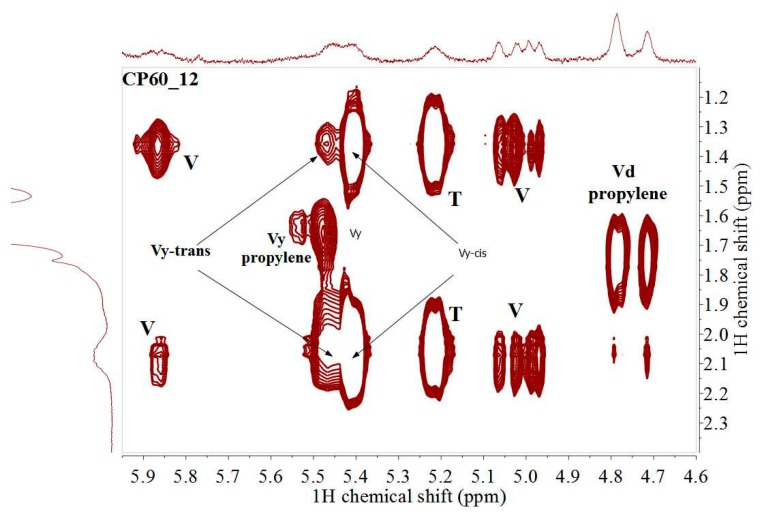
^1^H-^1^H TOCSY map of the sample C9PP60_12.

Comparisons made in Figure 7 between copolymers and their corresponding polyC_9_ samples really show that no *Vd* signals associated with C_9_ units are present. Supporting this assertion, the ^1^H-^1^H TOCSY spectrum of a copolymer (CPP60_12) shows correlation peaks between vinylidene signals and *Vd* methyl protons at 1.75 ppm (see in Figure 9).

Regarding vinyl chain-ends, they emerge according to trends shown in the upper plot of Figure 8, where it is quite noticeable that the progress is increasingly faster as *T*_pol_ decreases, i.e., when the selectivity of the catalyst/comonomer interaction is improved and the stability of the propagating catalyst/growing chain complex is expected to increase.

**Figure 10:**
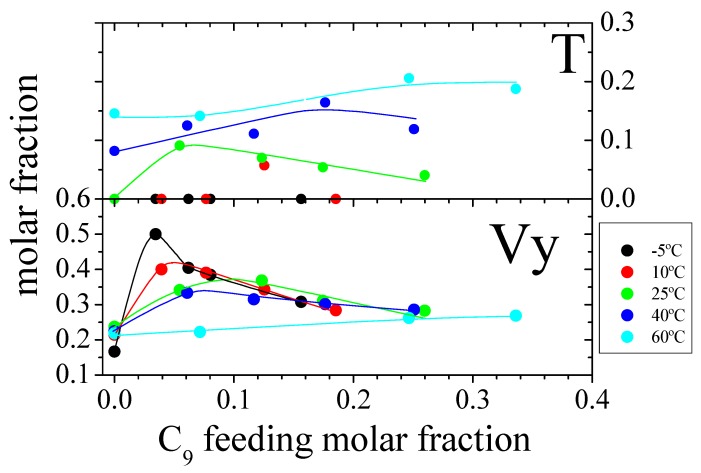
Evolution of *V*_y_ and T contents with the C_9_ feeding molar fraction for the different series.

Other emerging olefin species are vinylenes and trisubstituted olefins, of which their contents evolve with the C_9_ feeding, as depicted in Figure 10. While the former is present in all copolymer series, the latter is only found in copolymerizations at 40 °C upwards.

An inspection of ^1^H NMR spectra of PP and poly-1-nonene samples proves that *V*_y_ are characteristic of both homopolymers (see for instance bottom and top spectra in Figure 2). Although characteristic ranges of *V*_y_ protons coming from propene and C_9_ overlap quite a lot, those ones belonging to propene insertions display a low-field component, which allows the distinguishing of their existence. Once again, comparison of both homopolymers in Figure 2 illustrates this fact. Additionally, the TOCSY spectrum of the C9PP60_12 in Figure 9 proves the existence of propene derived *V*_y_ groups, since the distinctive correlation signal with the methyl peak at (5.54, 1.64 ppm) is observed.

According to this assignment, the *V*_y_ trends shown in the bottom plot of Figure 10 are ascribed to the increasing impact of *V*_y_ associated with C_9_ units. This is an expected fact, which is accompanied by a significant qualitative change in the signal with *T*_pol_. While a low-field multiplet is found in copolymers obtained at –5, 10, and 25 °C, another additional high-field multiplet is detected in series 40 and 60 (see Figure 7). This change is not related to C_9_ content, since the presence of one or two signals is preserved all along the range of the comonomer content studied at each series, and it must be connected to the temperature dependent production of one or two possible stereoisomers. The low-field signal is attributed to the trans configuration and the high-field component corresponds to the cis one [64,67].

Finally, trisubstituted olefins were detected at medium and high *T*_pol_. As a matter of fact, the signal at 5.22 ppm is exclusive of samples obtained from 25 °C upwards in PP, poly-1-nonenes, or copolymers at any comonomer content.

**Figure 11:**
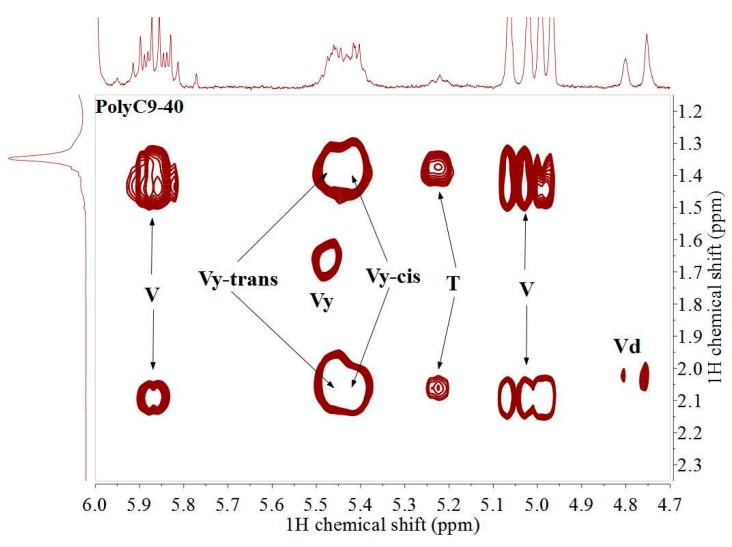
^1^H-^1^H TOCSY map of the sample polyC_9_40.

The structure was found to be very simple, as displayed by a triplet centred at 5.22 ppm, based on the ^1^H NMR spectra of PP and poly-1-nonene (bottom and top spectra in Figure 2, respectively). Such signal corresponds to the proton of a trisubstituted olefin contiguous to a methylene [68,69]. The TOCSY spectrum of the polyC_9_40 (Figure 11) reveals correlation signals of the *T* proton with methylene protons relatively placed in α and β, which are consistently assigned to such a structure.

The upper plot in Figure 10 displays the evolution of *T* olefins with the C_9_ feeding at the different *T*_pol_. The trends make evident that both C_9_ content and temperature promote them and, in particular, that the content turn out to rise from zero up to a maximum at a low comonomer content for the 25 °C series. This fact allows assuring that *T* olefins are entirely associated with C_9_ insertions in this case. On the contrary, the presence of significant contents in PP40 and PP60 leads us to presume that these structures have their origin from both propene and C_9_ insertions. Regardless, the increment with comonomer content is indicative of the increasing engagement of C_9_ units in *T* production. In fact, the absence of the *T* correlation signal with methyl protons in the 1.55–1.70 range (see TOCSY map in Figure 9) allows us to assure that most T olefins are produced from 2,1 insertions of C_9_.

## 4. Discussion

It is rather clear from Figure 4 that interaction of the catalyst site with C_9_ comonomer is increasingly favoured at low temperatures, i.e., under conditions promoting stable thermo-dynamic intermediates over those kinetically preferred. This fact has important consequences from a basic standpoint, since it reveals that such a kind of selectivity might play a considerable role in the catalyst′s activity [19,57] through changes in both initiation and termination of copolymerization.

Accordingly, the results of relative activities, shown in Figure 5, may be argued in similar terms. The raise in the relative activity and its shifting towards lower values of the C_9_ feeding molar fraction, when *T*_pol_ diminishes, must be related to the above-mentioned selectivity of the catalyst towards C_9_, rather than with the growing chain microstructure characteristics. As a matter of fact, the specific C_9_ content at which the maximum activity occurs differs from one set to another. C_9_ feeding fractions for the activity maxima do not correspond to similar C_9_ contents into the copolymers, but to chain compositions increasingly richer in C_9_ as *T*_pol_ rises up. Additionally, microstructure in tacticity was found to be quite different between the copolymer series. The tacticity distribution at the pentad level, shown in supplementary information (Table S1 in Supplementary Materials), displays that propene isotacticity decreases along with the rise in *T*_pol_, contributing this way to enhance the solubility of chains. Therefore, it can be concluded that relative selectivity of the active centre itself on both comonomers is a factor fully involved in the enhanced kinetics of the copolymerization, more so than other factors promoting mass transfer, like enhanced chain solubility. Therefore, this result argues against the dependence of the catalyst activity on the degree of polymer chain bundling, since variation of relative activities is higher the lower the *T*_pol_ used, just when the melting temperature increases (Table 1) and solubility of copolymers decreases.

Consequently, the selectivity of the catalyst-comonomer interaction must be a driving factor of the comonomer effect [19,57]. Firstly, the α-olefin plays as a ligand of the metal centre and this interaction [10,19], which is favoured at lower temperatures, might assist the separation of the ionic pair promoting an enhancement of the propagation rate. Secondly, and more importantly, the content of initiating sites available is expected to increase due to the role of the 1-nonene in the chain termination. This fact would be conclusive if a triggering effect of the comonomer in the chain initiation [4,12,44] is accepted. Indeed, the evolution of molecular weights, presented in Figure 6, provides strong evidence about the implication of 1-nonene in chain termination. The temperature dependence of the molecular weight goes along with the described variation of the catalyst specificity when interacting with C_9_. The analysis of chain-end groups, shown next, will allow the confirmation that C_9_ insertions are directly responsible for the termination processes and, thereby, for the recovery of chain-free active sites.

**Scheme 3:**
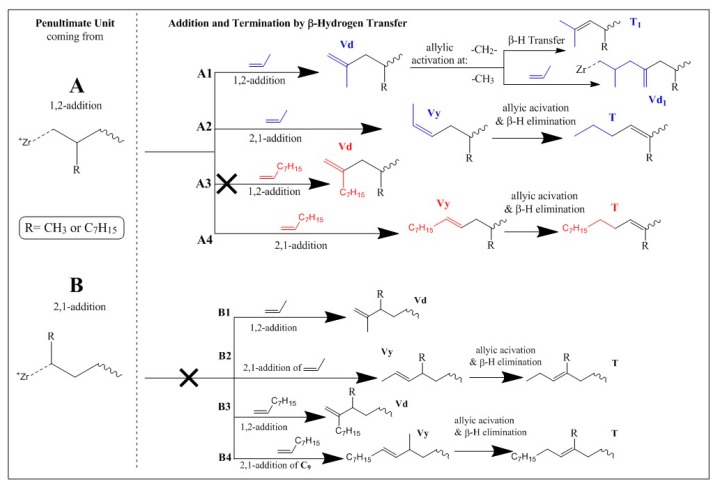
Termination routes (**A** and **B**) through the β-Hydrogen transfer processes.

**Scheme 4:**
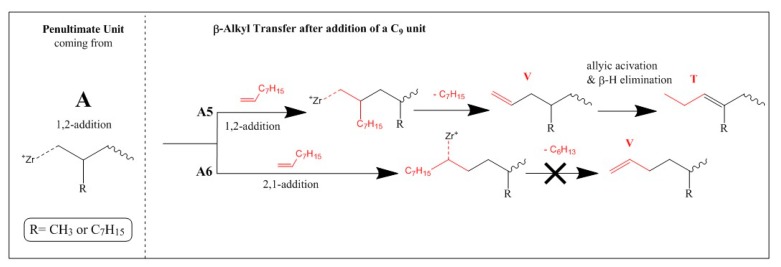
Termination route (**A**) through β-alkyl transfer processes from C_9_ end units.

The following discussion will be carried out on the assumption that termination mechanisms leading to chain-end olefins mainly involve transfer processes to the catalyst from the β position into the last inserted unit of growing chains. This ensemble of reactions has proven to successfully explain the presence of olefin species found in the copolymers under study [57,64,68,70,71,72,73,74,75,76,77,78]. Additionally, allylic activation of chain-end olefin species will also be considered in order to account for the formation of in-chain double bonds. The structures of chain-end groups are collected in Scheme 3 and Scheme 4. The former displays the olefin species resulting from β-H transfer processes and the latter displays those coming from β-alkyl transfers. Steric considerations lead to the exclusion of any possibility of insertion after the regio-irregular incorporation of either comonomers (routes B). Subsequent β-transfer steps would yield olefin structures that are not observed. The only exception is the well-known regular 1,2-propene addition after an irregular propene insertion, which produces mid-chain regiodefects detected in PP homopolymers and low C_9_ content copolymers.

**Vinylidenes** (*Vd*). The most important fact concerning the nature of these species is that *Vd* are exclusively caused by β-H transfers from regio-regular propene terminated chains (Scheme 3, route A1) because route A3 is forbidden. Absence of *Vd*, arising from regio-regular C_9_ co-units, is surprising if it is considered that this process is an effective termination mechanism in C_9_ homopolymerization.

The main alternative to this selective blocking towards the capture of the β-placed hydrogen in 1,2-inserted C_9_ units by the catalyst seems not to be propagation, but β-alkyl transfer, which will be discussed below. Whatever the reason might be, it is quite evident that the hindrance of a C_7_ long side branch itself is not the primary cause, since such a termination mechanism is not forbidden in poly-1-nonenes. The fact that *T*_pol_ is essential for the production of *Vd* chain-ends in some of the poly-1-nonenes (see the vanishing of *Vd* signals in polyC_9_ spectra in Figure 7 when *T*_pol_ is reduced), this suggests that more stable conformations in the catalyst-last linked unit structure are far from enabling short-range interactions between the metal centre and the hydrogen β-placed. A requirement that is necessary for the H-transfer to take place is through either a bimolecular or a unimolecular pathway [64].

Although the influence of *T*_pol_ on the feasibility of the β-H transfer is clear, it is indeed the conformational mobility of the chain that seems to be the true driving factor. This is supported by the unequivocal involvement of chain composition. Actually, it is apparent that rather rigid propene-rich chains in the C9PP60 series do not show any trace of C_9_-associated *Vd* groups, as would otherwise have been expected at 60 °C. It is then plausible that a minimum C_9_ content exists that is able to confer enough chain mobility to allow β-H transfers in regularly inserted C_9_ units.

**Figure 12:**
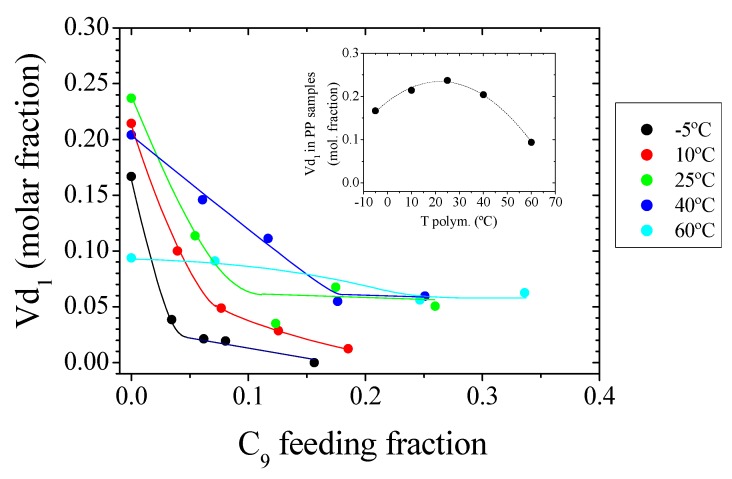
Evolution of *Vd*_1_ content with the C_9_ feeding molar fraction for the different series.

There is a particular type of *Vd* olefin that is mostly located inside chains (*Vd*_1_), as commented in the results section. These olefins are detected in both PP and copolymers and are exhibited by the increased intensity of the 4.79 ppm peak over the 4.71 ppm one [64,65,66,69,78]. These groups are directly associated with propene units and their presence has been justified by resorting to allylic re-activation of the *Vd* methyls (Scheme 3, pathway A1). This is a process that enters into competition with allylic activation of *Vd* methylene groups (also shown in Scheme 3, pathway A1), which renders the special trisubstituted olefin (*T*_1_). Nevertheless, the presence of C_9_ co-units greatly reduces the occurrence of *Vd*_1_ at any temperature. This is visually shown in Figure 12. In the specific case of PP samples, the variation of the relative content of *Vd*_1_ with *T*_pol_ displays the trend depicted in the insert of Figure 12. These groups decrease markedly at high temperatures, although its content seems to grow a little from –5 to 25 °C.

**Vinyls** (*V*). These chain-end olefins must come from β-alkyl transfers of the seven carbon length side branch in 1,2-inserted C_9_ units. Scheme 4 shows the range of possibilities in the generation of vinyls from regular insertion of C_9_ units. Among the options displayed, β-alkyl transfer is thought to be restricted to specific 1,2-inserted C_9_ units (route A5), since coordination states following 2,1-insertions of C_9_ have very accessible H atoms on both side β positions, which are available to be captured by the metal centre (route A6). Consequently, 2,1-inserted C_9_ units will evolve to vinylene functionalities, as shown in route A4 of Scheme 3.

When the selectivity of the catalyst/comonomer interaction is improved and the stability of the propagating catalyst/growing chain complex increases, i.e., at low *T*_pol_ values, then β-alkyl transfers are facilitated. The fact is that β-H transfer is absolutely blocked under any of the polymerization conditions used and the β-alkyl transfer becomes the only way out to finish propagation from 1,2-inserted C_9_ units, with the only option of further evolving to trisubstituted olefins (Scheme 4, route A5). This eventuality is disabled at –5 and 10 °C, as will be discussed below. To the best of our knowledge, the prevalence of such a termination mechanism in copolymerization is a novel result in α-olefin copolymerization.

As aforementioned in the discussion on vinylidenes, the selectivity of 1,2-inserted C_9_ units towards β-alkyl transfer is barely altered at 60 °C and for a C_9_ content of 12 mol %, despite the fact that β-H transfer in 1,2-inserted C_9_ shows up as an active mechanism in C_9_ homopolymerization. It is then clear that, even though *T*_pol_ is a factor to take into account the production of *Vd* associated with C_9_ units, the absence of these species in these copolymer samples results in suspicion that the composition of the growing chain has a significant role in the competition between both β transfer processes. This is an issue that is worth studying by widening the range of comonomer content.

**Vinylenes** (*V*_y_). It is assumed that these olefins have their origin in 2,1-insertions, whatever the unit inserted is, either propene [64,68,77,79] or α-olefin [70,71,72], and their formation can consistently be accounted for by routes A2 and A4 in Scheme 3.

According to the assignment detailed in the results section, it is evident that unimolecular β-H transfers in 2,1-inserted C_9_ units are the sole process when *T*_pol_ is –5, 10, and 25 °C, since only trans *V*_y_ groups are detected. However, at 40 and 60 °C, the copolymers also exhibit the high-field component, ascribed to the cis configuration, which must be related to the concurrence of bimolecular β-H transfers [64,67].

It is worth mentioning that when the C_9_ presence in the reaction medium rises up, the content of *V*_y_ apparently decreases in the series at –5 and 10 °C (Figure 10 down). This can be ascribed to a lesser impact of C_9_ 2,1-misinsertions since, as will be discussed in the next point, no further evolution of *V*_y_ to T species takes place at these temperatures. On the contrary, from 25 °C upwards, isomerization of *V*_y_ groups to T ones occurs to some extent. In these cases, the joint evolution of both olefins in copolymer samples shows little variation with the C_9_ content. Furthermore, no significant trend differences are found between them (see Graph S1 in Supplementary Materials).

**Trisubstituted olefins** (*T*). These species are thought to come from allylic activation of *V*_y_ groups [64,68,69,70]. The mechanism proposed in literature to account for T olefins is based on the fact that trisusbstituted and *V*_y_ species are found together in PP [64,68,70]. It assumes that allylic activation of *V*_y_ groups is the route leading to *T* olefins (pathways A2 and A4 in Scheme 3 and A5 Scheme 4). This mechanism seemingly plays a more active role when *T*_pol_ increases.

**Isobutyl ones** (*T*_1_) are a particular group within these chain-end olefins. These species are exclusively present in PP samples and they have been proposed to come from the isomerisation of *Vd* groups, via allylic activation through the interaction with the catalyst centre (Scheme 3, pathway A1) [64,68,69,78,79,80]. There is absolutely no evidence of this group in copolymers and further, the possibility of generating *T*_1_ via pathway A1 is ruled out in these samples. This fact additionally assures that such an isomerisation route does not occur as a consequence of the high temperature used in NMR characterisation, but because of termination mechanisms.

**Figure 13:**
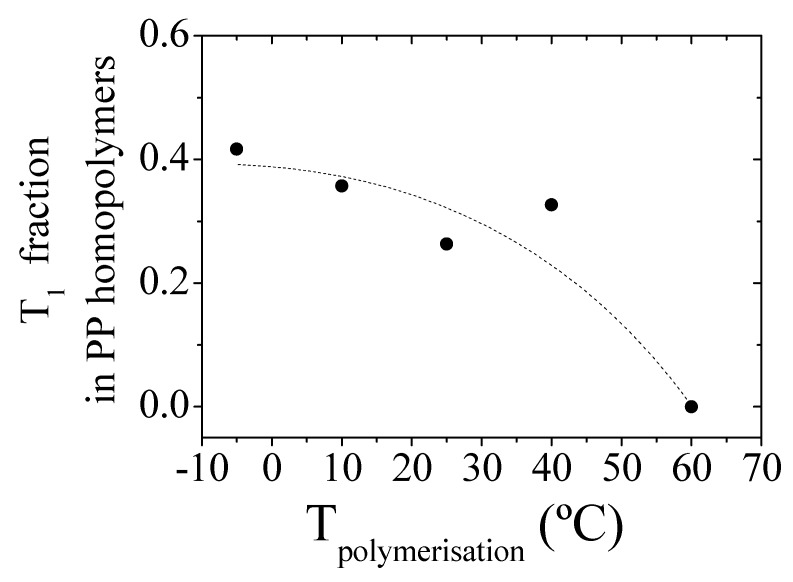
Evolution of T_1_ content in PP samples with *T*_pol_.

The relative content of *T*_1_ in PP samples is found to decrease with *T*_pol_, following the trend shown in Figure 13. Such an evolution can be reasonably explained in terms of the ability of the catalyst centre to interact back with the vinylidene, an eventuality that is expected to be promoted at low temperatures when rather slow termination kinetics favour the interaction between the catalyst and the closest olefin *Vd* group.

It is, therefore, apparent that termination events associated with C_9_ insertions are directly responsible for the molecular weight reduction found and, hence, for the recovery of free-chain catalyst centres. The mechanistic considerations performed allow the identification of which termination routes lay behind the chain termination ability of inserted C_9_ units. While regio-irregular insertions necessarily lead to β-H transfer, regio-regular ones show a high propensity to finish chain propagation through β-alkyl type. The former produces vinylene chain-ends and the latter, vinyls. Such processes are in fact the main source of free-chain active sites during copolymerization, of which their involvement in the triggering action of the α-olefin has been suggested as the origin of the “comonomer effect” [44]. This work′s results point to that possibility, however the way that it would occur is open to discussion, because the specificity of the metallocene catalysis may require the trigger mechanism, proposed by Ystenes [12] for Ziegler-Natta catalysts, to be adapted. In fact, the solvation effect reviewed by Kryzhanovskii [19] might serve as the α-olefin assisted mechanism to explain rate enhancement.

It has been proposed that comonomer molecules themselves play a role as transfer agents, causing molecular weight decrease [7]. Although this possibility of producing chain-free catalyst sites exists, this work shows how fresh initiating sites mostly come from the very chain releasing mechanisms associated with C_9_ insertions, even without the engagement of any other molecule. In fact, β-alkyl transfer is probably unimolecular [81,82] and β-H transfer at –5 and 10 in 2,1-C_9_ insertions are also entirely unimolecular, as can be deduced from the exclusive formation of trans-*V*_y_ olefins [64,67].

## 5. Conclusions

The metallocene copolymerization of propene and 1-nonene is a temperature dependent process. The selectivity of the catalyst towards the α-olefin is, in fact, higher as the polymerization temperature is lowered. This fact seems to lay at the core of the activity increase undergone by the catalyst, just by adding small fractions of 1-nonene. Accordingly, the relative activity increment turns out to be proportional to the selectivity for the 1-nonene insertion.

The α-olefin promotes the termination of propagation by means of chain transfer mechanisms, from both regio-regular and regio-irregular insertion modes. As a matter of fact, although 1,2-insertions of C_9_ do not necessarily yield chain termination, the β-alkyl transfer process is specially preferred and vinyl chain-ends derived from this mechanism are predominant all along the conversion range studied. Concurrently with this termination pathway, regio-irregular 2,1-insertions also contribute significantly, since its occurrence stops chain growth by forcing β-Hydrogen transfer to take place. In this case, the resulting chain-end olefins are vinylidene type.

The detailed transfer processes furnish chain-free active sites, which have been suggested to be involved in especially promoted insertions, mediated by the α-olefin′s triggering role. These results agree with some recent findings on Phillip catalysis and open up a new insight into the comprehension of the comonomer effect in metallocene copolymerization.

Finally, it is worth highlighting that the absolute prevalence of β-alkyl transfer over β-Hydrogen one in 1,2-inserted C_9_ units is a non-reported selective termination mechanism, of which its underlying reasons must still be addressed. The fact that both termination events take place in C_9_ homopolymerization in a relative proportion that depends on *T*_pol_ suggests that competence between the two processes is likely governed by chain mobility.

## Figures and Tables

**Table 1:** Characteristics of polymerization runs and samples.

Sample	*T*_pol_^a^ (°C)	C_9_ Feeding (Molar Fraction)	Time (min)	Activity ^b^	^13^C NMR r_1_.r_2_^c^	C_9_ Content (mol %)	*M*_n_ ^1^H NMR (SEC) (g·mol^−1^)	*T*_m_^d^ (°C)
PP−5	−5	0	167	284	--	0	(38,700)	151.0
C9PP−5_2		0.035	51	789	0	2.0	34,900	127.0
C9PP−5_4		0.062	10	2882	0	4.2	19,800	110.0
C9PP−5_5		0.080	12	3396	0.5	4.8	18,000	104.5
C9PP−5_6		0.099	13	1866	0	6.2	--	95.5
C9PP−5_8		0.156	82	757	1.4	8.0	12,500	84.0
PP10	10	0	25	2266	--	0	38,200 (39,200)	149.0
C9PP10_2		0.039	20	3521	0	2.3	32,600	123.5
C9PP10_4		0.077	26	1267	0	4.0	23,300	110.5
C9PP10_7		0.126	9	17,779	0.5	6.6	14,000	92.5
C9PP10_9		0.185	13	8530	1.5	9.3	12,400	75.0
PP25	25	0	28	2517	--	0	29,000 (34,500)	145.5
C9PP25_2		0.055	17	5240	0	2.3	22,500	120.5
C9PP25_5		0.123	5	19,792	1.2	5.1	16,800	99.0
C9PP25_8		0.174	41	2273	2.2	8.2	14,200	88.5
C9PP25_11		0.260	74	2250	1.1	11.2	10,800	66.0
PP40	40	0	6	18,213	--	0	21,500 (21,400)	141.5
C9PP40_2		0.061	5	30,454	0	2.2	21,300	120.0
C9PP40_4		0.117	6	24,356	0	4.0	18,800	105.0
C9PP40_7		0.176	8	55,478	1.9	7.4	13,800	85.5
C9PP40_9		0.251	8	19,222	1.5	9.4	12,700	72.0
PP60	60	0	3	42,201	--	0	8600	131.0
C9PP60_2		0.072	5	30,271	0	2.4	9700	112.5
C9PP60_5		0.159	3	50,077	0	5.1	--	97.0
C9PP60_8		0.160	19	6862	1.4	7.6	9600	78.5
C9PP60_12		0.336	16	12,928	1.4 (1.3) ^e^	11.9 (12.2) ^e^	6800	54.5

(a) Temperature of polymerization; (b) Activity units are: Kg·mol^−1^_[Zr]_ mol^−1^_[Prop]_·h^−1^; (c) Values of zero for the reactivity ratio product correspond to the exclusive presence of isolated C_9_ unit; (d) Values obtained by Differential Scanning Calorimetry (DSC); (e) Values obtained from a ^13^C NMR spectrum with a 10 s delay time.

## Supplementary Materials

The following are available online at https://www.mdpi.com/2073-4360/11/8/1266/s1, Table S1: Relative content of pentads and regiodefects (in mol %) and average isotactic length (*n*_1_), Graph S1: Joint evolution of *V*_y_ and *T* olefins with the C_9_ feeding molar fraction.
