# Supplementary material for: A New Insight into the Comonomer Effect through NMR Analysis in Metallocene Catalysed Propene–co–1-Nonene Copolymers
Source: Polymers (Basel). 2019 Jul 31;11(8):1266. doi: 10.3390/polym11081266 (PMC6723554; doi:10.3390/polym11081266)
Supplement: Supplementary file 1 [file polymers-11-01266-s001.pdf]

Supplementary materials

A New Insight into the Comonomer Effect through NMR Analysis in Metallocene Catalysed Propene–*co*–1-Nonene Copolymers

Qiong Wu [1], Alberto García-Peñas [2,3], Rosa Barranco-García [4], María Luisa Cerrada [4], Rosario Benavente [4], Ernesto Pérez [4] and José Manuel Gómez-Elvira [4,*]

1 School of Engineering, Hong Kong University of Science and Technology, Clear Water Bay, 999077 Kowloon, Hong Kong;
2 College of Materials Science and Engineering, Shenzhen Key Laboratory of Polymer Science and Technology, Guangdong Research Centre for Interfacial Engineering of Functional Materials, Nanshan District Key Laboratory for Biopolymers and Safety Evaluation, Shenzhen University, Shenzhen 518060, China;
3 Key Laboratory of Optoelectronic Devices and Systems of Ministry of Education and Guangdong Province, College of Optoelectronic Engineering, Shenzhen University, Shenzhen 518060, China;
4 Instituto de Ciencia y Tecnología de Polímeros (ICTP-CSIC), Juan de la Cierva 3, 28006 Madrid, Spain;
* Correspondence: elvira@ictp.csic.es; Tel.: (34)912-587-524

Graph S1. Joint evolution of Vy and T olefins with the C_9 feeding molar fraction.

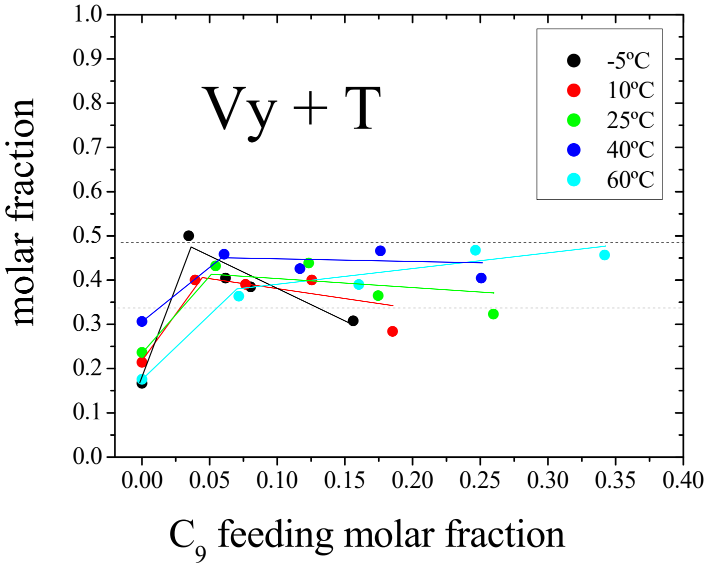

Table S1. Relative content of pentads and regio-defects (in mol %) and average isotactic length (n_1)[a]

Sample	mmmm	mmmr	rmmr	mmrr	mmrm + rmrr	mrmr	rrrr	mrrr	mrrm	Regio-defects	n_1[a]
PP-5	89.9	4.8	1.5	2.6	0	0	0	0	0.5	0.7	47
CP-5_2	93.6	2.8	0	2.3	0	0	0	0	0.9	0.4	24
CP-5_4	94.3	3.0	0	1.8	0	0	0	0	0.5	0.4	17
CP-5_5	94.3	2.5	0.3	1.8	0	0	0	0	0.7	0.4	15
CP-5_6	94.7	5.3	0	0	0	0	0	0	0	0	13
CP-5_8	92.7	3.1	0.6	1.8	0.7	0	0	0	0.6	0.5	10
PP10	90.8	3.5	1.5	2.4	0.2	0	0	0	0.6	1.0	42
CP10_2	93.6	2.5	0.2	2.0	0.7	0	0	0	0.6	0.4	23
CP10_4	92.6	2.9	0.2	2.2	1.1	0	0	0	0.7	0.3	15
CP10_7	92.2	2.9	0.6	2.1	0.7	0	0	0	0.8	0.7	11
CP10_9	92	4.3	0.3	2.1	0.8	0	0	0	0.5	0	9
PP25	87.7	4.2	2.7	3.3	1	0	0	0	0.9	0.2	31
CP25_2	92.1	2.8	0.3	2.4	0.8	0	0	0	0.9	0.7	19
CP25_5	90.3	3.7	0.9	2.7	1.0	0	0	0	0.9	0.5	12
CP25_8	89.5	4.2	1.1	2.9	0.9	0	0	0	0.8	0.6	10
CP25_11	90.2	4.5	1.2	2.4	1.0	0	0	0	0.7	0	8
PP40	86.2	4.8	2.2	4.0	1.2	0	0	0	1.1	0.5	25
CP40_2	89	3.9	0.5	3.2	1.1	0	0	0	1.2	1.1	17
CP40_4	90.1	3.7	0.6	2.9	1.0	0	0	0	1.2	0.5	14
CP40_7	88.5	4.4	0.8	3.2	1.3	0	0	0	1.3	0.5	9
CP40_9	88.7	4.7	0.8	2.7	1.5	0	0	0	0.9	0.7	8
PP60	75.3	9.8	2.8	7.8	2.4	0	0	0	1.9	0	14
CP60_2	82.5	7	0	5	2.5	0	0	0	2.4	0.6	12
CP60_5	82.6	7.6	0	5.0	2.8	0	0	0	2.0	0	9
CP60_7	85	6.5	0	4.2	2.5	0	0	0	1.8	0	8
CP60_12	82.1	7.0	1.7	4.2	2.8	0	0	0	1.7	0.5	6
CP60_12[b]	82.4	7.2	1.6	4.0	2.7	0	0	0	1.7	0.4	6

[a]Average isotactic length: $n_1 = \frac{[mm]+[mx]}{^1/_2[mx]}$ (with "m" meso propylene unit and "x" either racemic or regiodefect or C_9 unit);
[b]Regio and stereo-microstructure calculated from ^{13}C NMR spectrum performed with a 10 s delay time instead of 5 s
